# *Momordica charantia* (Bitter Melon) Reduces Obesity-Associated Macrophage and Mast Cell Infiltration as well as Inflammatory Cytokine Expression in Adipose Tissues

**DOI:** 10.1371/journal.pone.0084075

**Published:** 2013-12-17

**Authors:** Bin Bao, Yan-Guang Chen, Lei Zhang, Yan Lin Na Xu, Xin Wang, Jian Liu, Wei Qu

**Affiliations:** School of Biotechnology and Food Engineering, Hefei University of Technology, Hefei, China; McGill University, Canada

## Abstract

Obesity is a world-wide epidemic disease that correlates closely with type 2 diabetes and cardiovascular diseases. Obesity-induced chronic adipose tissue inflammation is now considered as a critical contributor to the above complications. *Momordica charantia* (bitter melon, BM) is a traditional Chinese food and well known for its function of reducing body weight gain and insulin resistance. However, it is unclear whether BM could alleviate adipose tissue inflammation caused by obesity. In this study, C57BL/6 mice were fed high fat diet (HFD) with or without BM for 12 weeks. BM-contained diets ameliorated HFD-induced obesity and insulin resistance. Histological and real-time PCR analysis demonstrated BM not only reduced macrophage infiltration into epididymal adipose tissues (EAT) and brown adipose tissues (BAT). Flow cytometry show that BM could modify the M1/M2 phenotype ratio of macrophages in EAT. Further study showed that BM lowered mast cell recruitments in EAT, and depressed pro-inflammatory cytokine monocyte chemotactic protein-1 (MCP-1) expression in EAT and BAT as well as interleukin-6 (IL-6) and tumor necrosis factor-α (TNF-α) expression in EAT. Finally, ELISA analysis showed BM-contained diets also normalized serum levels of the cytokines. In summary, in concert with ameliorated insulin resistance and fat deposition, BM reduced adipose tissue inflammation in diet-induced obese (DIO) mice.

## Introduction

Obesity is a world-wide epidemic disease that correlates closely with many metabolic abnormalities, such as type 2 diabetes and cardiovascular diseases [1-3]. During the initiation and progression of diet-induced obesity, along with body weight gain, various inflammatory cells, including macrophages [4,5], mast cells [5,6] and lymphocytes [7,8] infiltrate into adipose tissues. Their infiltration disturbs the balance among the immune cells in adipose tissues, for example the ratio of “classically activated” (M1-type) *versus* “alternatively activated” (M2-type) macrophages was elevated [9]. Thus in obese individuals, the adipose tissues possess hypertrophic adipocytes and more inflammatory cells and produce more pro-inflammation mediators, including cytokines, chemokines and hormones [10]. The mediators either assemble in or leak out of adipose tissues, leading to the enhancement of inflammation levels in local tissues and/or sera. Finally, the pathological events further exacerbate fat deposition and obesity-related insulin resistance [10,11]. Although the mechanisms are not sufficiently clear, obesity-associated adipose tissue inflammation has been regarded as an important contributing factor to the above metabolic diseases [10,11].

*Momordica charantia* (bitter melon, BM) is a popular nutritious and healthy vegetable in Asian countries, and it is also used as traditional anti-diabetes and anti-obesity medicine in these areas [12,13]. In the past few years, the beneficial effects of BM or its extracts on obesity and obesity-associated insulin resistance were continuously affirmed in various experimental animals, including mice [14-20] and rats [21-23]. The related mechanistic study indicated that BM or its constituents might enhance AMP-associated protein kinase (AMPK) [14,15], peroxisome proliferator activating receptors (PPARs) [16,17] and insulin [18-21] signals in tissues, reduce lipogenic gene expression in adipose tissues [22], and increase lipid oxidation in adipose tissues [23]. With regard to obesity-associated inflammation, dietary BM can suppress pro-inflammatory mediator leptin and resistin levels in adipose tissues [16] and plasma [16,20], elevate system levels of anti-inflammatory mediator adiponectin [17] and improve system and brain inflammation [24] in mice fed with high fat diets (HFD). However, it is little known whether BM could reduce the levels of adipose tissue inflammation, including inflammatory cells and cytokines, in diet-induced obese (DIO) mice. Therefore, study of BM anti-inflammation mechanism is necessary and meaningful.

In this study, we investigated the effects of BM on adipose tissue inflammatory cell infiltration and cytokine expression caused by obesity. BM-contained diets ameliorated HFD-induced obesity and insulin resistance. Meanwhile, BM not only reduced macrophage infiltration into EAT and BAT, but also modified the M1/M2 phenotype ratio of macrophages in these tissues. Further study showed that BM lowered mast cell recruitments in EAT, and depressed pro-inflammatory cytokine monocyte chemotactic protein-1 (MCP-1) expression in EAT and BAT as well as interleukin-6 (IL-6) and tumor necrosis factor-α (TNF-α) expression in EAT. Finally, BM-contained diets also normalized serum levels of the cytokines. In summary, BM ameliorated insulin resistance and fat deposition, and reduced adipose tissue inflammation in DIO mice.

## Materials and Methods

### BM

Fresh BM fruits were purchased from the local market. The sample was identified by Prof. Zhou Zhong Ze from School of Resources and Environmental Engineering, Anhui University, China and a voucher (NO. MC20110924) is deposited in Herbarium of School of Biotechnology & Food Engineering, Hefei University of Technology, China. According to Huang et al. [22], BM fruits were washed with tap water, and then cut into small pieces, freeze-dried at -40°C, followed by powdering and stored at -20°C. 

### Mice

Five-week-old male C57BL/6 mice were purchased from Vital River Laboratory Animal Technology Co. Ltd. (Beijing, China). Hefei University of Technology Standing Committee on Animals approved all animal protocols. All mice were housed in ventilated cages within a pathogen-free barrier facility that maintained a 12-hour light/12-hour dark cycle and allowed free access to autoclaved water and irradiated food. To examine the effect of BM on diet-induced obesity and diabetes, the mice were randomly divided into 6 treatment groups: group 1, were fed on HFD (n=8); group 2, were fed on HFD supplemented with 2% BM (n=8); group 3, were fed on HFD supplemented with 5% BM (n=8); group 4, were fed on low food diet (LFD) (n=8); group 5, were fed on LFD supplemented with 2% BM (n=8); group 6, were fed on LFD supplemented with 5% BM (n=8). HFD and LFD were based on Research Diets D12451 diet (45 kcal% fat) and D12450B diet (10 kcal% fat) respectively. The detailed contents of all 6 diets were shown in Table 1. The body weights and food intakes of mice were measured at the same time of every week. At 17-week-old, the mice were sacrificed by CO_2_, and their sera and related fat tissues, including EAT, SAT and BAT were harvested and stored at -80 °C. 

**Table 1:** The composition of diets used in experiment^1^.

Ingredient (g)	HFD	HFD+2%BM	HFD+5%BM	LFD	LFD+2%BM	LFD+5%BM
Casein	200	200	200	200	200	200
L-Cystine	3	3	3	3	3	3
Corn starch	72.8	62.03	45.9	315	301.76	282.03
Maltodextrin 10	100	100	100	35	35	35
Sucrose	172.8	172.8	172.8	350	350	350
Cellulose	50	43.61	34	50	42.14	30.22
Soybean oil	25	25	25	25	25	25
Lard	177.5	177.5	177.5	20	20	20
Mineral Mix 10026	10	10	10	10	10	10
Dicalcium phospate	13	13	13	13	13	13
Calcium carbonate	5.5	5.5	5.5	5.5	5.5	5.5
Potassium citrate	16.5	16.5	16.5	16.5	16.5	16.5
Vitamin Mix V10001	10	10	10	10	10	10
Choline bitartrate	2	2	2	2	2	2
Freeze-dried BM **^2^**	0	17.16	42.9	0	21.1	52.75
Total weight (g)	858.1	858.1	858.1	1055	1055	1055
Total energy (kcal)	4057	4057	4057	4057	4057	4057

1. High fat diet (HFD) was based on the Research Diets D12451 diet, and low fat diet (LFD) was based on the Research Diets D12450B diet.

2. BM was added in diets as freeze-dried full bitter melon powers.

### Insulin tolerance tests and glucose tolerance tests

According to standard protocol [6], insulin tolerance tests (ITT) and glucose tolerance tests (GTT) were performed before the mice were sacrificed. Briefly, to carry out ITT or GTT, fasted overnight mice were injected intraperitoneal with insulin (1.5U/kg bodyweight, WanBang BioPharma) or glucose (1g/kg bodyweight, Sigma) respectively. The blood samples were taken from mice tail tip. Blood glucose concentrations were measured by a blood glucose meter (Omnitest Plus, B.BRAUN, Kyunggi, Korea) at the time before injection and 15 min, 30 min, 45 min, 60 min, 90 min, 120 min after injection. 

### Histology and immunohistochemistry

Harvested tissue specimens were fixed in 4% formalin for 24 hours at room temperature, embedded in paraffin and cut into five micron thick sections. All tissue sections were baked at 80°C for 20 min and de-paraffin in xylene at 50°C for 30 min and rehydrated in a graded ethanol series. To test adipocyte sizes, macrophage positive area and the numbers of mast cells in adipose tissues, we performed haematoxylin-eosin staining, immunohistochemistry with anti-Mac-2 monoclonal antibody (1:400, BioLegend) and toluidine blue staining, respectively. For measurement of macrophage positive area, 5 random fields each section were evaluated by detecting staining intensity with the Image-Pro Plus Version 6.0 (Media Cybernetics, Inc, Shanghai, China) and data were presented as positive cells area percent (%) in the tissues. Mast cell numbers in each section were counted under a light microscope (OPTEC), and data were presented as cell numbers per mm^2^.

### RNA extraction and quantitative real-time PCR

Total RNA was extracted from frozen tissues (100 mg) using TRIzol reagent (TaKaRa, Dalian, China). The cDNAs were synthesized using superscript III reverse transcriptase and oligo d(T)18 primers according to manufacturer’s protocol (Invitrogen). Real-time quantitated PCR was performed using the power SYBR green mix (TaKaRa) as previously described [25]. The primer sequences of all tested genes were listed in Table 2. 

**Table 2:** Primers for quantitative real-time PCR.

Gene	Sequence of forward primers(5' to 3',)	Sequence of reverse primers(5' to 3',)
*F4/80*	CTTTGGCTATGGGCTTCCAGTC	GCAAGGAGGACAGAGTTTATCGTG
*Itgax*	CTGGATAGCCTTTCTTCTGCTG	GCACACTGTGTCCGAACTC
*Inos*	CCAAGCCCTCACCTACTTCC	CTCTGAGGGCTGACACAAGG
*Chi3l3*	AGAAGGGAGTTTCAAACCTGGT	GTCTTGCTCATGTGTGTAAGTGA
*Cd163*	CCTGGATCATCTGTGACAACA	TCCACACGTCCAGAACAGTC
*mMcp-6*	GCCCAGCCAATCAGCG	CCAGGGCCACTTACTCTCAGA
*Mcp-1*	CCCCAAGAAGGAATGGGTCC	GGTTGTGGAAAAGGTAGTGG
*Il-6*	CCTTCCTACCCCAATTTCCAA	AGATGAATTGGATGGTCTTGGTC
*Tnf-α*	ACGGCATGGATCTCAAAGAC	AGATAGCAAATCGGCTGACG
*Leptin*	GAGACCCCTGTGTCGGTTC	CTGCGTGTGTGAAATGTCATTG
*Ucp-1*	CACTCAGGATTGGCCTCTACG	GGGGTTTGATCCCATGCAGA

### Stromal vascular fraction (SVF) isolation and Flow cytometry

EAT from male C57BL/6 mice were excised and minced in PBS. Tissue suspensions were centrifuged 400g for 5 minutes to remove free leukocytes. Then tissue was incubated in 2 mg/ml collagenase (Sigma-Aldrich) at 37 °C for 30 minutes with shaking. A 75-μm filter were used to remove adipocyte. After a spin at 400 g for 5 minutes, SVF pellet were collected in the bottom. The SVF pellet was resuspended in PBS, then RBC Lysis Buffer was added and incubated for 5 minutes. After washing by PBS, cells were incubated with Fc Block (BD Biosciences, San Diego, USA) for 15 minutes on ice [9]. Then antibodies against F4/80 -APC, CD11b- PerCP-Cy5.5, CD11c-PE, CD206-FITC (eBioscience, San Diego, USA) were added, and incubated for 30 minutes on ice followed by 2 washes in PBS. Then cells were analyzed by FACS (FACSVerse; BD Biosciences). Data were analyzed on FlowJo software. 

### ELISA

The serum levels of IL-6, TNF-α, MCP-1, leptin and insulin were measured using corresponding mouse ELISA kits (BD Bioscience, San Diego, USA) according to manufacturer’s protocols.

### Statistical analysis

All data were present as means ± SEM, and the difference between groups was assessed by Mann-Whitney test owing to our small data size and non-normal distribution data *P* < 0.05 was considered statistically significant. 

## Results

### Bitter melon ameliorated fat deposition and insulin resistance

**Figure 1:**
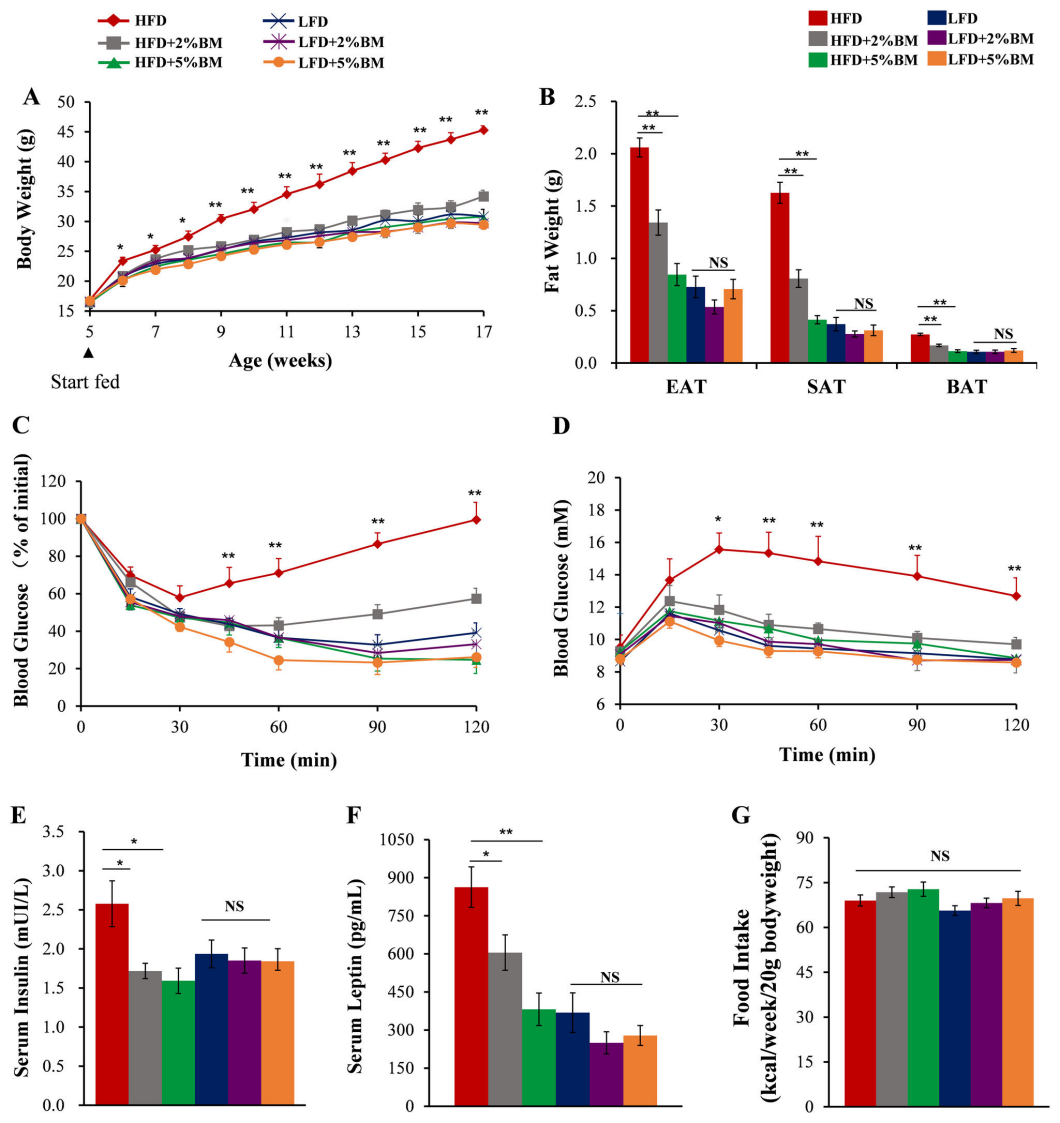
Bitter melon ameliorated fat deposition and insulin resistance in mice fed with HFD. Five-week-old male C57BL/6 mice were grouped and fed on the diets indicated in Table 1. (A) Body weight gain of the mice. (B) The weights of epididymal adipose tissues (EAT), subcutaneous adipose tissues (SAT) and brown adipose tissues (BAT) of the mice. (C) Insulin tolerance and (D) glucose tolerance assays were performed at 17-week-old in the mice. The insulin (E) and leptin (F) concentrations in serum of mice. (G) The energy intake per 20g bodyweight of mice during 12-week treatment. Statistical difference between groups is shown using a nonparametric Mann-Whitney test. n=8 per group. *P<0.05, **P<0.01. All data are means ± SEM.

**Figure 2:**
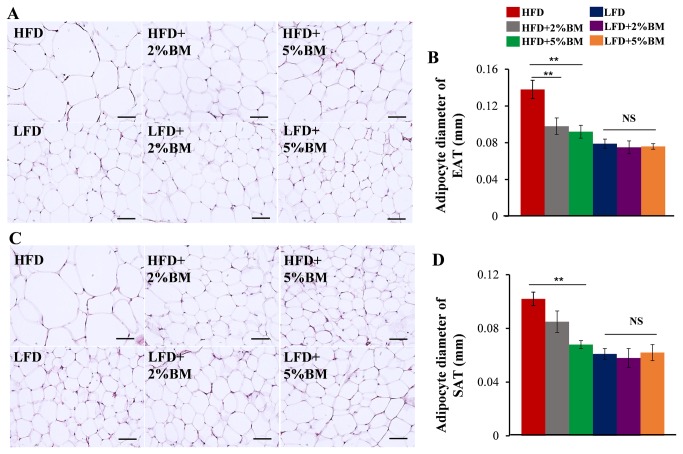
Bitter melon reduced adipocyte size in mice fed with HFD. HE staining of EAT (A) and SAT (C) in mice, scale bars are 50 μm in the length. (B) Quantification of EAT adipocyte diameter and (D) SAT adipocyte diameter. (Data were collected from H&E-stained sections from eight individual mice, five fields per mouse, using Image-Pro Plus 6.0 software) Statistical difference between groups is shown using a nonparametric Mann-Whitney test. n=8 per group. *P<0.05, **P<0.01. All data are means ± SEM.

To induce obesity or test the effects of BM on obesity, we fed the mice with HFD or HFD supplemented with 2% or 5% BM for 12 weeks, and the mice fed with LFD or LFD contained the same percent BM were used as controls (detailed composition of diets are shown in Table 1). As we expected, HFD led to a higher body weight gain in mice compared with LFD (Figure 1A). The increased trend in body weight gain was attenuated sharply in mice fed on HFD supplemented with either 2% or 5% BM, while there was no similar body weight reduction occurred among 3 groups of mice fed with LFD (Figure 1A). To clarify the possible mechanism that BM inhibited body weight gain in mice with HFD, we tested food intakes per week of mice and determined their fat weights and adipocyte sizes in EAT, SAT, and BAT at 17-week-old. The results showed that BM-contained diets had no effects on food intakes of mice (Figure 1G), but 2% or 5% BM supplement significantly reduced all tested adipose tissue weights (Figure 1B) and adipocyte sizes (Figure 2) in mice with HFD. Such an effect was not observed in mice fed with LFD (Figure 1B and Figure 2). Along with lower body weights, the mice fed on BM-contained HFD had improved insulin sensitivity (Figure 1C) and glucose tolerance (Figure 1D) compared with those mice fed only on HFD. Correspondingly, serum insulin and leptin (Figure 1E and 1F) levels were reduced in the mice.

### Bitter melon inhibited the recruitments of inflammatory macrophages into EAT and BAT

Adipose tissue inflammation is a key hallmark of obesity and obesity-associated insulin resistance [10,11]. To test the impact of dietary BM on adipose tissue inflammation, we firstly examined the distribution of macrophages in EAT, SAT and BAT.

**Figure 3:**
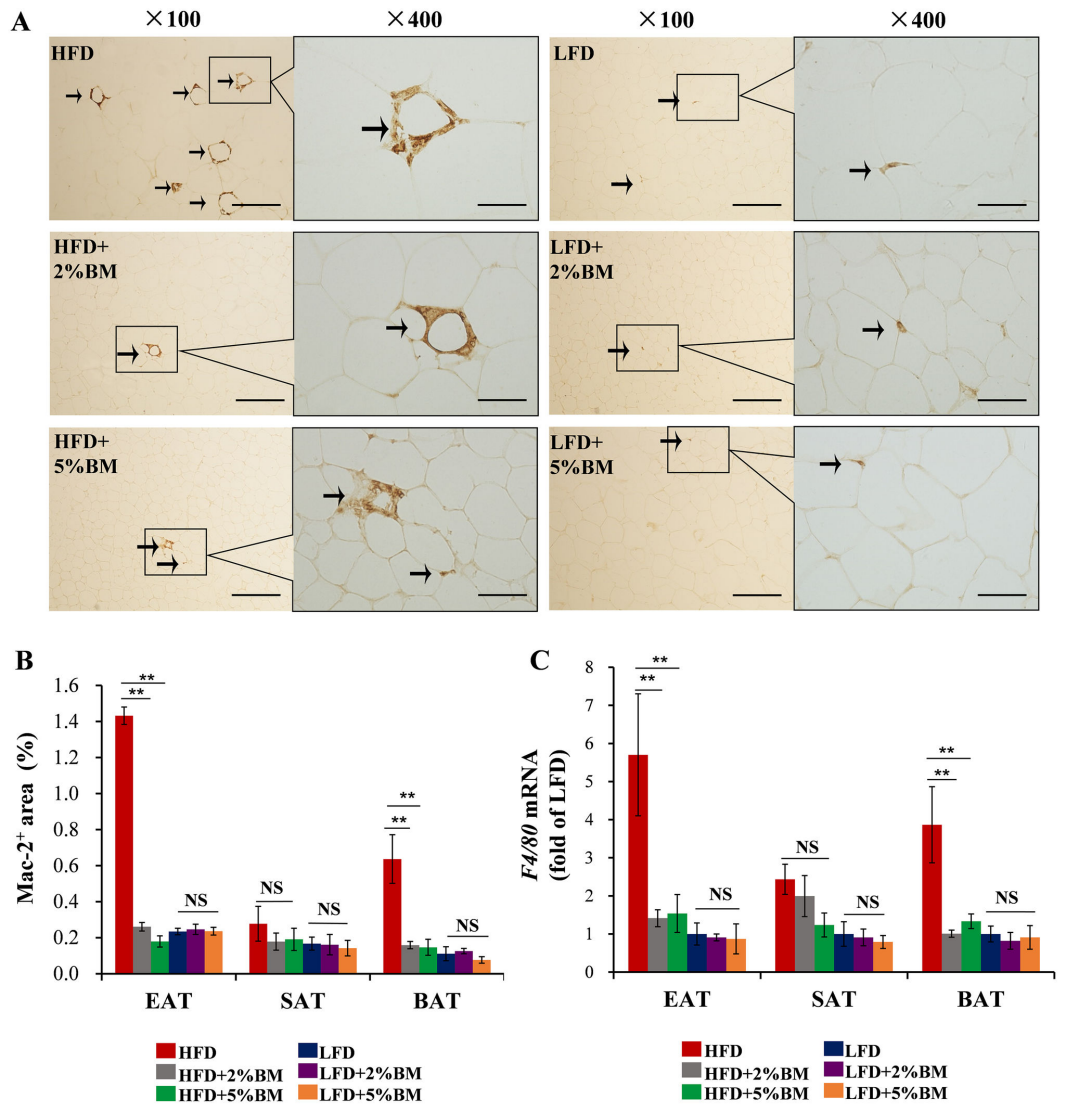
Bitter melon inhibited the recruitments of inflammatory macrophages into EAT and BAT in mice. (A) Mac-2 immunostaining in EAT of mice; arrows indicate Mac-2^+^ macrophages; scale bars in (A), 100μm in length in low power views (×100) and 25μm in length in high power views (×400). (B) Quantification of Mac-2^+^ macrophage area in EAT, SAT and BAT of mice. (C) Real-time PCR quantitative mRNA expression of macrophage marker gene *F4/80* in EAT, SAT and BAT of mice. Statistical difference between groups is shown using a nonparametric Mann-Whitney test. n=8 per group. *P<0.05, **P<0.01. All data in (B)-(C) are means ± SEM.

**Figure 4:**
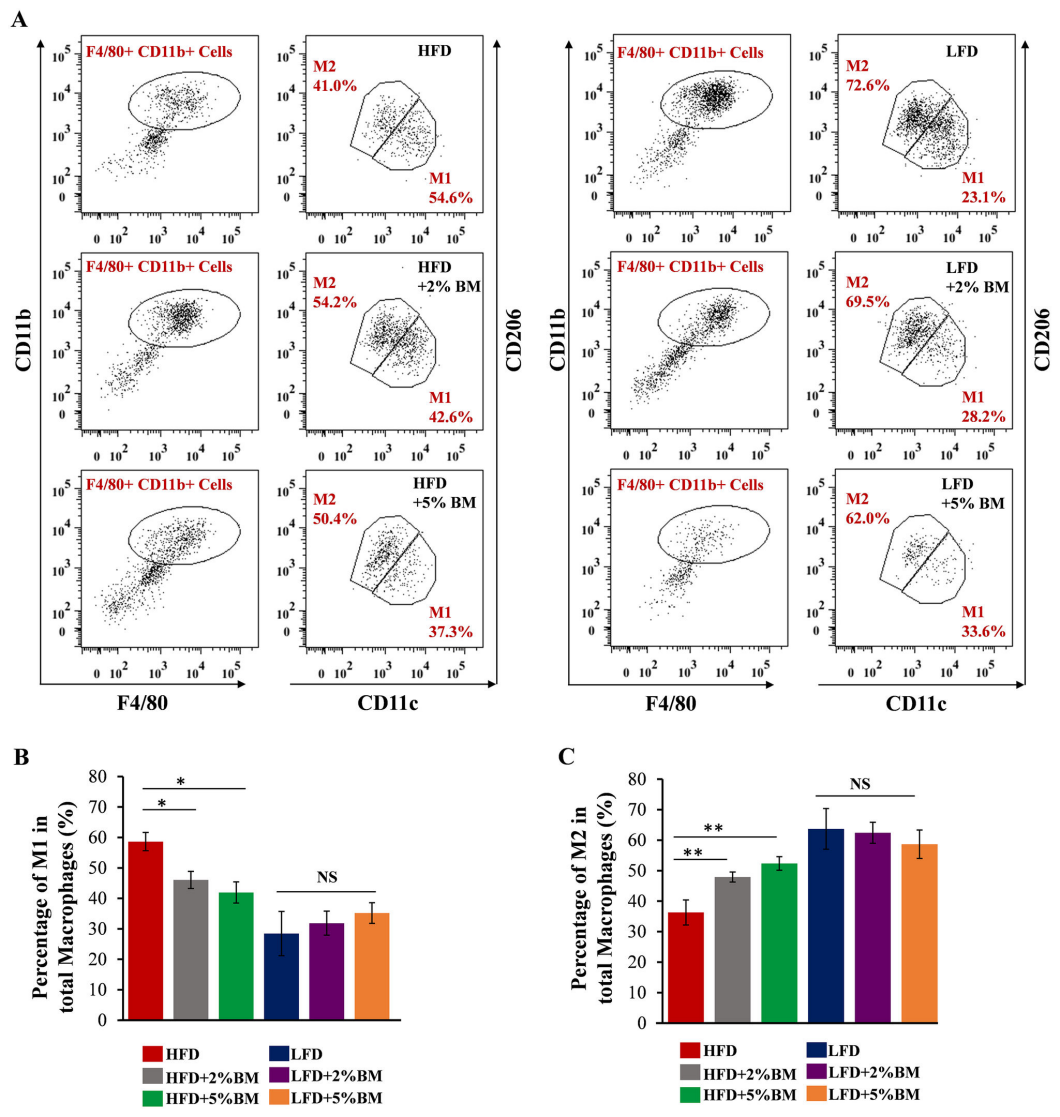
BM induced phenotypic switch in macrophage polarization in EAT of mice. (A) Analysis of SVF cells for M1 and M2 macrophage. SVF cells were stained with antibodies against F4/80, CD11b, CD11c, CD206, and analyzed by flow cytometry. Samples were gated for F4/80+ CD11b+ cells, then examined for coexpression of CD11c or CD206. M1 and M2 macrophage and their percentage were indicated in red. (B and C) Quantitation of percentage for M1 and M2 macrophage in total F4/80+ CD11b+ cells. Statistical difference between groups is shown using a nonparametric Mann-Whitney test. n=6 per group. *P<0.05, **P<0.01. All data in (B)-(C) are means ± SEM.

**Figure 5:**
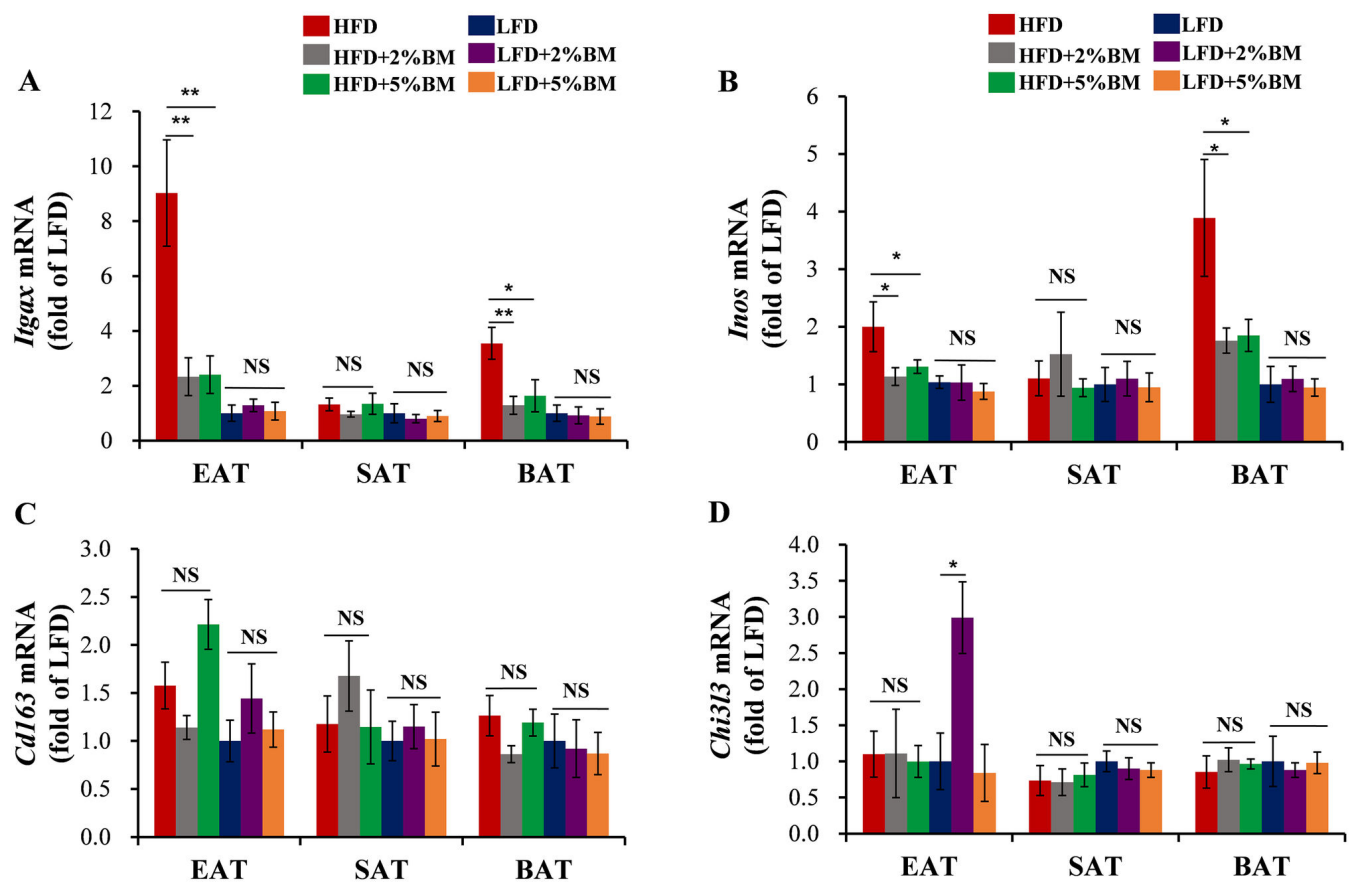
BM inhibited subset change of macrophages in EAT and BAT of mice. The M1 marker gene expression of macrophages *Itgax* (A) and *Inos* (B) in EAT, SAT and BAT of mice. The M2 marker gene expression of macrophages *Cd163* (C) and *Chi3l3* (D) in EAT, SAT and BAT of mice. Statistical difference between groups is shown using a nonparametric Mann-Whitney test. n=8 per group. *P<0.05, **P<0.01. All data in (A)-(D) are means ± SEM.

Immunohistochemistry staining with monoclonal antibody Mac-2 detected significantly larger macrophage positive corona-areas in EAT (Figure 3A and 3B) and BAT (Figure 3B), but not in SAT (Figure 3B), from obese mice fed with HFD than those from lean mice fed with LFD. BM-contained diets reduced potently the trend in mice with HFD (Figure 3A and 3B). Expression of *F4/80*, the common marker of macrophage, is consistent with the immunohistochemistry results (Figure 3C). Mutlicolour flow cytometry was used to confirm the subsets of macrophages in EAT. BM induced a phenotypic switch in macrophage polarization in EAT (Figure 4). BM-contained diets significantly decreased the high proportion of M1 that caused by HFD (Figure 4B), and relatively increased the proportion of M2 in total macrophages in EAT (Figure 4C). Further, we investigated the expression of *Itgax* and *Inos* (specific markers for M1 macrophage), and *Cd163* and *Chil3l* (specific markers for M2 macrophage) [9,26]. BM-contained diets also significantly reduced mRNA expressions of *Itgax* (Figure 5A) and *Inos* (Figure 5B) in EAT and BAT of mice with HFD, but mRNA expressions of *Cd163* (Figure 5C) and *Chil3l* (Figure 5D) were not affected in these mice. The results imply BM-contained diets not only inhibit the recruitments of macrophages into EAT and BAT, but also modify the unnatural ratio of M1/M2 in these adipose tissues in mice with HFD. 

### Bitter melon decreased the numbers of mast cells in EAT

**Figure 6:**
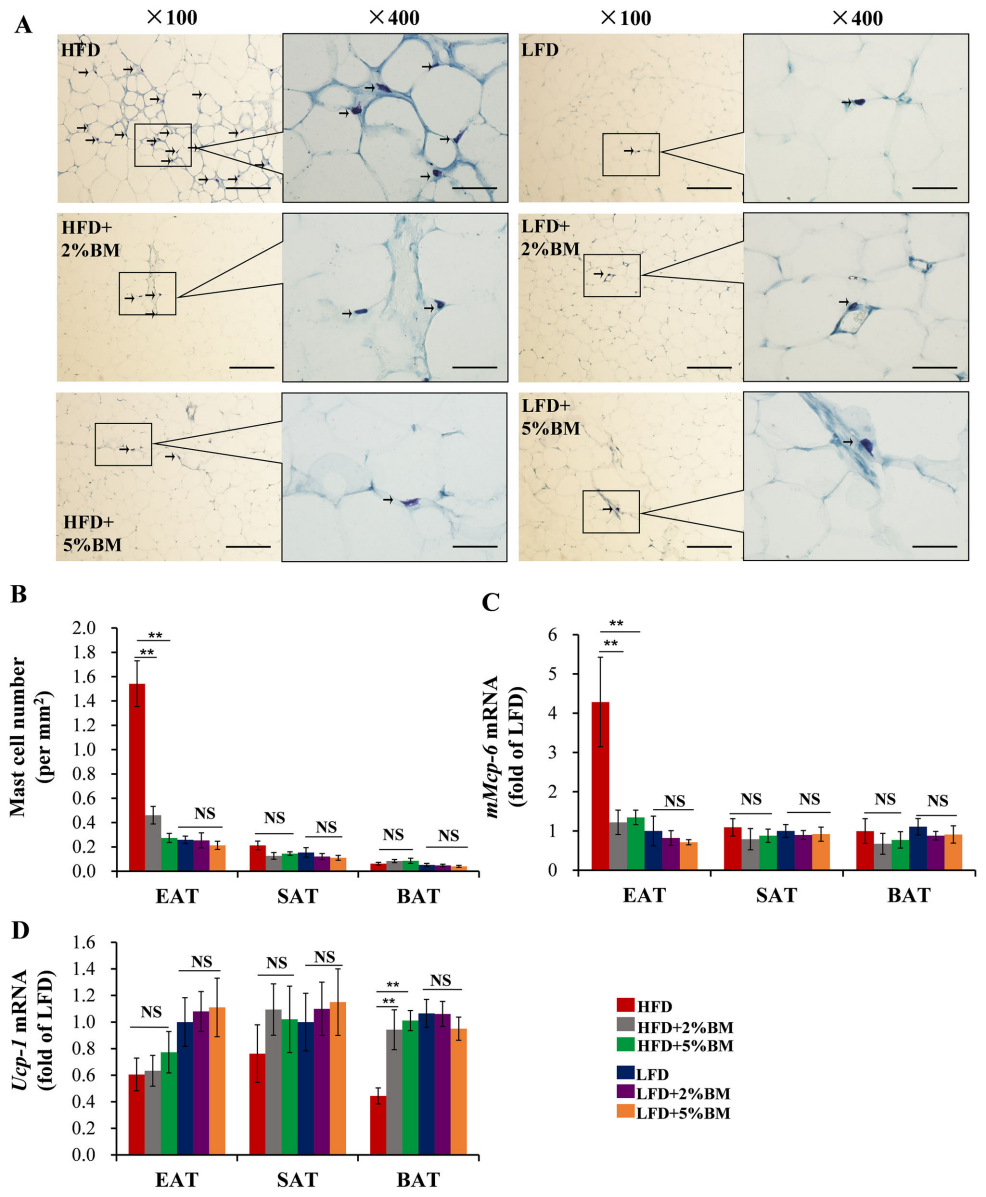
BM inhibited recruitment of mast cells in EAT of mice. (A)Toluidine blue staining of mast cells in EAT of mice; arrows indicate toluidine blue staining mast cells; scale bars in (A), 100μm in length in low power views (×100) and 25μm in length in high power views (×400). (B) The quantification of the numbers of mast cells in EAT, SAT and BAT of mice. (C) The expression of mast cell marker gene m*Mcp-6* in EAT, SAT and BAT of mice. (D) The expression of energy expenditure marker gene *Ucp-1* in EAT, SAT and BAT of mice. Statistical difference between groups is shown using a nonparametric Mann-Whitney test. n=8 per group. *P<0.05, **P<0.01. All data in (B)-(D) are means ± SEM.

Mast cell is a crucial inflammatory cell in diet-induced obesity and diabetes [6,11]. Our pervious study reported that there were more mast cells in EAT in DIO mice than those in lean mice. By contributing to mouse adipose tissue cysteine protease cathepsin expression, apoptosis and angiogenesis, the cells are involved in diet-induced obesity and glucose intolerance [6]. Using toluidine blue staining, we found that HFD induced an increased mast cell recruitment in EAT (Figure 6A and 6B), but not in SAT and BAT (Figure 6B). BM-contained diets suppressed the mast cell number enhancements in EAT of the mice with HFD, while there was no significantly difference among 3 groups with LFD (Figure 6A and 6B). Correspondingly, BM-contained diets also depressed the expression of mast cell maker gene *mMcp-6* in EAT (Figure 6C). We reported previously that genetic deficiency and pharmacological stabilization of mast cells can increase energy expenditure and promote UCP-1 protein expression in BAT [6]. Consistent with the previous results, the mice fed on HFD with 2% or 5% BM had an up-regulated *Ucp-1* mRNA expression in BAT (Figure 6D), along with their less mast cell numbers in EAT.

### Bitter melon reduced inflammation cytokine levels in adipose tissues and sera

**Figure 7:**
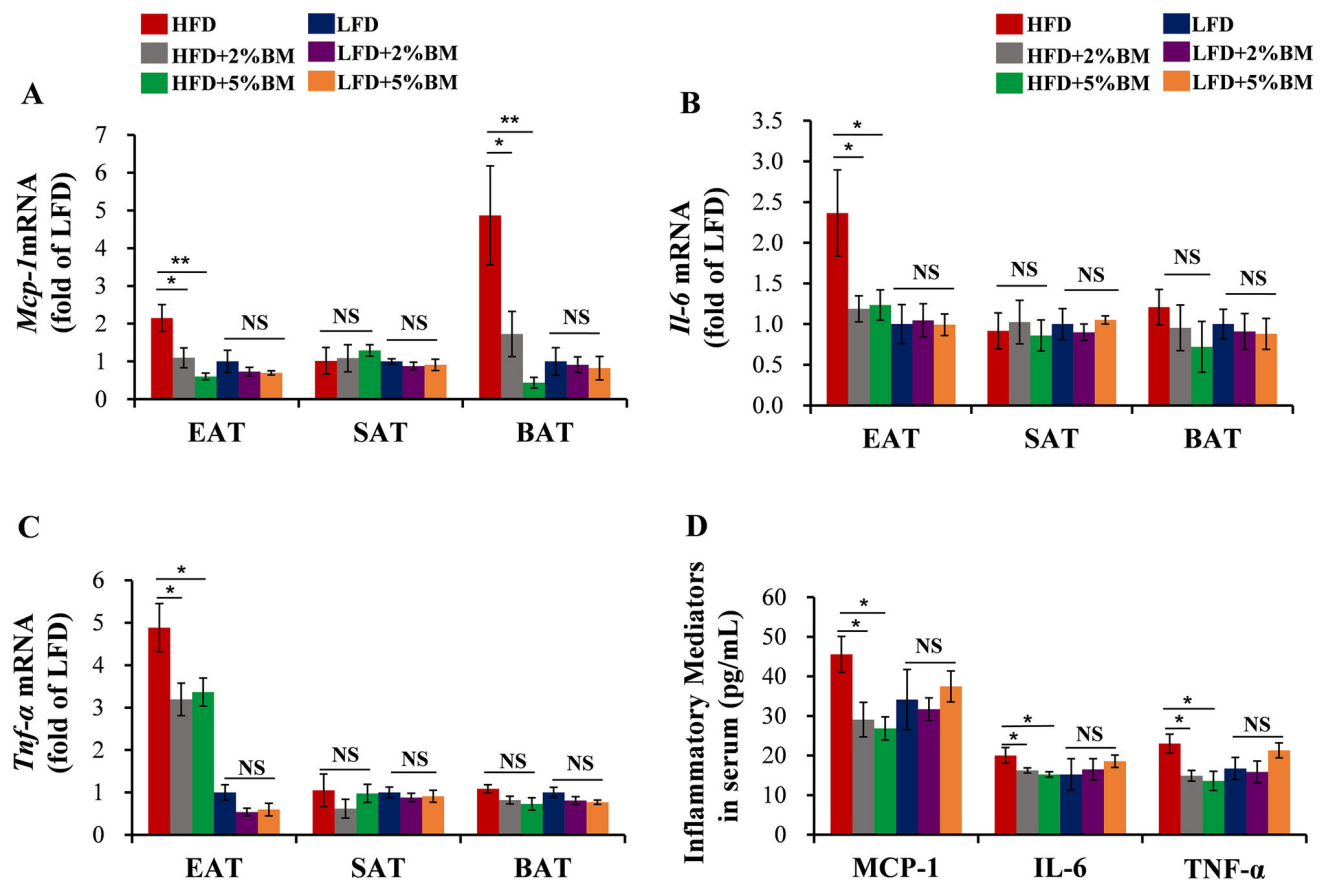
BM down-regulated inflammation mediator levels in adipose tissues and serum of mice. The mRNA expression of *Mcp-1* (A)*, Il-6* (B) and *Tnf-α* (C) in EAT, SAT and BAT of mice. (D) The inflammation mediator concentrations in serum were measured by mouse MCP-1, IL-6 and TNF-α ELISA kit of mice. Statistical difference between groups is shown using a nonparametric Mann-Whitney test. n=8 per group. *P<0.05, **P<0.01. All data in (A)-(D) are means ± SEM.

To assess the effects of BM on the increased local inflammatory levels in DIO mice, we measured the mRNA expression levels of several important pro-inflammatory cytokines involved in fat deposition and insulin resistance [10], including *Mcp-1, Il-6* and *Tnf-α* in EAT, SAT and BAT. Real-time PCR showed HFD led to increased *Mcp-1* expression in EAT and BAT (Figure 7A)*, Il-6* (Figure 7B) and *Tnf-α* (Figure 7C) expressions in EAT and the increased expressions were suppressed by BM-contained diets in mice with HFD. And then, we tested the serum levels of these cytokines using ELISA assays, which revealed the mice fed on HFD with 2% or 5% BM had a similar reduction in the serum inflammatory mediator concentrations (Figure 7D). These results suggest that BM-contained diets suppress adipose tissue and system inflammation levels, and may hereby improve fat deposition and insulin resistance in DIO mice. 

## Discussion

BM is a traditional and functional vegetable for several metabolic diseases, including obesity and diabetes [12,13], in which chronic adipose tissue inflammation is regarded as a major factor of impaired insulin sensitivity [10,11]. Previous reports showed that BM or its components could reduce insulin resistance and fat deposition accompanied by obesity in different experimental conditions [14-23]. Therefore, we hypothesized BM can reduce adipose tissue inflammation in DIO mice. To test the hypothesis, we reaffirmed the anti-obesity and anti-diabetes action of BM in DIO mice (Figures 1 and 2). Our results showed BM-contained diets could reduce HFD-induced body weight gain (Figure 1A) and related adipose tissue hyperplasia (Figure 1B and Figure 2), improve insulin resistance and glucose intolerance (Figure 1C and 1D), and lower serum levels of insulin and leptin (Figure 1E and 1F). 

In bodies of humans and rodents, there are various specific functional and locational adipose tissues, such as visceral adipose tissues, SAT and BAT [10,27]. Both visceral and subcutaneous adipose tissues are white adipose depots, but they are really distinct in obesity-associated inflammation. Responding to HFD, visceral adipose tissues, for example EAT, possess more inflammatory cell recruitments and secrete more abundant pro-inflammatory mediators than SAT [10,11]. While BAT play a critical role in energy expenditure as the primary thermogenesis regulator, HFD can induce BAT to exhibit more inflammatory and immune responses [28]. Since BM-contained diets can reduce diet-induced obesity and may hereby affect inflammation levels in these various adipose tissues, we studied a detailed inflammatory profile, including macrophages, mast cells and some pro-inflammatory cytokines in EAT, SAT and BAT.

Using anti-Mac-2 monoclonal antibody, we firstly performed immunohistochemistry analysis of adipose tissue macrophages, which have garnered the most attention in adipose tissue inflammation [10,11,29]. BM-contained diets significantly inhibited the infiltration of macrophages into EAT and BAT (Figure 3) in mice with HFD. It is well known that a macrophage sub-type shift from M2-polarized type to M1 pro-inflammatory type occurs in adipose tissue of experiment animals, along with diet-induced body weight gain and insulin resistance [9,26]. Therefore, using mutlicolour flow cytometry, we analyzed macrophage polarization in EAT. BM significantly induced a switch of M1 to M2 in EAT from mice fed with HFD (Figure 4). We also examined the expression of some macrophage or macrophage subtype specific genes in above mouse adipose tissues using real-time PCR. We found that the maker gene of total macrophage *F4/80* expression levels were lower in EAT and BAT in mice fed on BM-contained HFD than those in mice fed only on HFD (Figure 3C), supporting our above immunostaining using antibody to Mac-2 (Figure 3A and 3B). Moreover, the increased expression levels of M1 macrophage specific genes *Itgax* and *Inos* were reversed by BM-contained diets in EAT and BAT in mice with HFD (Figure 5A and 5B) and the expression levels of M2 macrophage specific genes *Cd163* and *Chil3l* were not changed in the tissues (Figure 5C and 5D). The data indicate that BM-contained diets reduce not only total macrophage distribution levels but also the sub-type radio of M1/M2 macrophages in EAT and BAT of DIO mice, which may protect those body weight reduced mice from enhanced adipose tissue inflammation. 

Once mast cells were known for their playing a critical role in immunoglobulin E–associated allergic disorders, recent studies show the inflammatory cells are involved in pathogenesis of metabolic diseases, such as obesity, diabetes and atherosclerosis [6,30,31]. We previously reported that there are more mast cells in EAT in DIO mice than those in lean mice and described the possible mechanisms that the cells promote diet-induced obesity and glucose intolerance [6]. Using toluidine blue histological analysis, we re-presented the HFD-induced mast cell number enhancement in EAT (Figure 6A and 6B). Further, we found that BM-contained diets significantly reduced the number of mast cells and the mRNA expression level of mast cell maker gene *mMcp-6* in EAT in mice fed with HFD (Figure 6A-6C). Interestingly, along with less mast cells, BM-contained diets up-regulated the mRNA expression of energy expenditure maker gene *Ucp-1* in BAT in mice fed on HFD (Figure 6D). The phenomena not only supported our pervious reports that genetic deficiency and pharmacological stabilization of mast cells enhanced energy expenditure in mice [6], but also suggested that BM-contained diets might ameliorate insulin resistance and fat deposition by suppressing mast cell infiltration and hereby improving energy expenditure in DIO mice. 

However, unlike BM-contained HFD, BM-contained LFD had no effect on physiology indexes (Figure 1 Figure 2) in mice, this may due to absent adipose inflammation in LFD-fed mice. This results supported that BM could reduce HFD-induced body weight gain through ameliorating adipose tissue inflammation in mice. When added to low fat diet, BM had no effect on macrophage (Figure 3) and mast cell (Figure 6) infiltration, and sub-type radio of M1/M2 macrophages (Figure 4 and Figure 5) in EAT, SAT and BAT. The results suggested that BM could reduce inflammatory cells infiltration and polarization in mice on HFD, but not affect the behaviors of inflammatory cells in healthy mice. 

Nerukar et al. recently reported that diets supplemented with freeze dried BM juice (BMJ) improved HFD-induced neuroinflammation [24]. They used cytokine antibody array to show that BMJ diet supplement reduced plasma levels of some important pro-inflammatory cytokines implicated in obesity-associated adipose tissue inflammation [10], such as IL-6, TNF-α and MCP-1 in mice with HFD. To examine whether whole BM fruit (WBF) diet supplement can impact adipose tissue inflammatory cytokine levels, we use real-time PCR to test the mRNA expression of *Mcp-1, Il-6* and *Tnf-α* in EAT, SAT and BAT. The results showed that BM-contained diets lower *Mcp-1* expression in EAT and BAT as well as *Il-6* and *Tnf-α* expression in BAT in the mice fed on HFD (Figure 7A-C). Further, ELISA assays indicated the lower levels of their corresponding protein in sera of the mice (Figure 7D). In adipose tissues, the pro-inflammatory cytokines, which were up-regulated by HFD and normalized by BM, can be expressed in both adipocytes and stromal vascular cells, including macrophages and mast cells. We can’t conclude which cells express and secrete the cytokines in adipose tissues, but BM-regulated adipose tissue inflammatory cell and pro-inflammatory cytokine reduction might be involved in improvement of insulin resistance and fat deposition in DIO mice.

Unlike our above results, a recent study showed that diets supplemented with BM seed (BMS) oil increased TNF-α concentration and macrophage crown-like structure number in EAT in mice with HFD, along with reduced body weight gain and unaffected serum glucose and insulin levels in the mice [32]. It seems that BMS contain more distinctive pro-inflammatory ingredients than other portion of BM fruits, while whole BM fruits preparation, such as WBF and BMJ, present a holistic anti-inflammatory characteristic, which may lead to the difference of BM fruits and BMS in biological function. Indeed, our here study and other studies have showed WBF [15,22] or BMJ [18,23,33,34] can effectively inhibit obesity and obesity-associated insulin resistance in DIO animals, while BMS preparation only exhibited one-sided effects on obesity [32] and blood glucose enhancement [35].

BM contains many active ingredients, including steroid and triterpenoid glycosides, alkaloids, flavonoids, polyphenols, carotenoids, fatty acids, and some active polypeptides (including insulin-like peptides) [36–38]. In these chemicals, the idiographic comments inhibit obesity-associated adipose tissue inflammation is not known, but some of them have showed potential related activities. Anti-diabetic activities of some triterpenoids from BM have been reported [14,39,40]. Although it isn’t clear whether their anti-diabetic actions have any correlations with obesity-associated adipose tissue inflammation, some hypoglycemic triterpenes have been showed an effective inhibitory activity to TNF-α-induced insulin resistance and inflammation in hepatic cell FL83B [41,42]. Moreover, a potent macrophage inflammation inhibitor [43] and mast cell stabilizer [44] quercetin were also found in BM fruits [24,45]. 

In summary, our results present the effects of BM on adipose tissue inflammation in DIO mice. BM reduced the adipocyte hyperplasia, recruitments of inflammatory M1-type macrophages and mast cells into EAT and/or BAT, might hereby suppress adipose tissue and system inflammation levels. Targeting special functional components of BM and their mechanism to regulate tissue and system inflammation may have therapeutic potential to prevent or reduce the development of obesity-associated complications, a hypothesis that merits further investigation in experimental animals and humans.
